# The effect of monohydroxyethylrutoside on doxorubicin-induced cardiotoxicity in patients treated for metastatic cancer in a phase II study

**DOI:** 10.1038/sj.bjc.6603994

**Published:** 2007-10-16

**Authors:** A M E Bruynzeel, H W M Niessen, J G F Bronzwaer, J J M van der Hoeven, J Berkhof, A Bast, W J F van der Vijgh, C J van Groeningen

**Affiliations:** 1 Department of Medical Oncology, 2 PK BR 010, VU University Medical Center, De Boelelaan 1117, Amsterdam 1081 HV, The Netherlands; 2 Department of Pathology, VU University Medical Center, Amsterdam 1081 HV, The Netherlands; 3 ICaR-VU, VU University Medical Center, Amsterdam 1081 HV, The Netherlands; 4 Department of Cardiac Surgery, VU University Medical Center, Amsterdam 1081 HV, The Netherlands; 5 Department of Cardiology, VU University Medical Center, Amsterdam 1081 HV, The Netherlands; 6 Department of Clinical Epidemiology and Biostatistics, VU University Medical Center, Amsterdam 1081 HV, The Netherlands; 7 Department of Internal Medicine, Ziekenhuis Amstelland, Amstelveen 1186 AM, The Netherlands; 8 Department of Pharmacology and Toxicology, Faculty of Medicine, University of Maastricht, Maastricht 6200 MD, The Netherlands

**Keywords:** monoHER, monohydroxyethylrutoside, doxorubicin, cardiotoxicity, clinical phase II study

## Abstract

The purpose of this study was to investigate the cardioprotective effect of the semisynthetic flavonoid 7-monohydroxyethylrutoside (monoHER) on doxorubicin (DOX)-induced cardiotoxicity in a phase II study in patients with metastatic cancer. Eight patients with metastatic cancer were treated with DOX preceded by a 10 min i.v. infusion of 1500 mg m^−2^ monoHER. Five patients were examined by endomyocardial biopsy after reaching a cumulative dose of 300 mg m^−2^. Histopathological changes in the cardiomyocytes (Billingham score) were compared with those described in literature for patients treated with DOX only. The mean biopsy score of the patients was higher (2.7) than the mean score (1.4) of historical data of patients who received similar cumulative doses of DOX. Although there is a considerable variability in few investigated patients, it was indicative that monoHER enhanced DOX-induced cardiotoxicity. However, the antitumour activity of DOX seemed better than expected: three of the four patients with metastatic soft-tissue sarcoma had a partial remission and the fourth patient stable disease. It is likely that the relatively high dose of monoHER is responsible for the lack of cardioprotection and for the high response rate in patients with soft-tissue sarcoma possibly by depleting the glutathione defense system in both heart and tumour.

The anthracycline doxorubicin (DOX) is widely used in the treatment of several malignancies in adult and paediatric patients. Treatment with DOX is limited by a dose-dependent cardiotoxicity, which may lead to late side effects resulting in severe morbidity and mortality (Steinherz *et al*, 2001; Lipshultz *et al*, 2005). Although the 5-year survival of childhood cancer has improved from 30 to 70% in the last 40 years, the risk of death from cardiac events in these survivors is eight times higher than that in the normal population (Wouters *et al*, 2005). Besides this, combining DOX with other anticancer drugs, for example, taxanes and trastuzumab, increases efficacy, but unfortunately also augments cardiotoxicity (Seidman *et al*, 2002; Minotti *et al*, 2004).

Although the mechanism of DOX-induced cardiotoxicity is still not fully understood, a major role has been ascribed to the induction of free radicals (Horenstein *et al*, 2000; Hrdina *et al*, 2000; Xu *et al*, 2001). The cardiomyocyte is particularly vulnerable to free radical injury because of properties such as a low antioxidant status (Doroshow *et al*, 1980; Iarussi *et al*, 2000). Presently, the cardioprotectant dexrazoxane is the only drug with proven efficacy (Cvetkovic and Scott, 2005). A recent review recommended the use of dexrazoxane if the risk of cardiotoxicity is high. However, clinicians should weigh its cardioprotective effect against the risk of a possible decrease of antitumour activity (Van Dalen *et al*, 2005). Preclinical experiments showed that the flavonoid 7-monohydroxyethylrutoside (monoHER) is a potential protective agent against DOX-induced cardiotoxicity without interfering with its antitumour activity (Van Acker *et al*, 1997, 2000). Radical scavenging and iron chelating properties are the supposed mechanisms of action of monoHER (Haenen *et al*, 1993; Van Acker *et al*, 1993). No serious side effects were observed in a clinical phase I study up to a dose of 1500 mg m^−2^. At this dose, the pharmacokinetic end points were reached, that is, *C*_max_ and AUC^∞^ were comparable to those obtained in mice under protecting conditions. Therefore, this dose was evaluated in a phase II study (Willems *et al*, 2006).

In the present study, we evaluated the cardioprotective properties of 1500 mg m^−2^ monoHER given as a 10 min i.v. infusion before DOX in patients with metastatic cancer. For the early sensitive and specific detection of DOX-induced cardiotoxicity, endomyocardial biopsies were taken (Meinardi *et al*, 1999).

## MATERIALS AND METHODS

### Patient selection

Patients with metastatic solid tumours were entered when they received a DOX-based chemotherapy regimen with a dosage of DOX⩾50 mg m^−2^ per cycle and an infusion duration ⩽1 h. Patients had a WHO performance status of ⩽2 and a life expectancy of ⩾3 months. They also had adequate organ functions. Their left ventricular ejection fraction (LVEF), measured by multigated radionuclide angiography, was >50%. Patients were excluded if they had received prior anthracyclines, had prior or actual cardiovascular disease or had prior radiotherapy to the mediastinum.

All patients gave written informed consent and the protocol was approved by the Medical Ethical Review Committee of the VU University Medical Center (VUMC). Patients were enrolled between September 2003 and March 2006.

### Treatment

7-Monohydroxyethylrutoside was provided by Novartis Consumer Health (Nyon, Switzerland). The drug was formulated by the Department of Pharmacy, VUMC, Amsterdam as described before (Willems *et al*, 2006). Formulated DOX (doxorubicin hydrochloride, 2 mg ml^−1^) was obtained from Pharmachemie B.V. (Haarlem, the Netherlands).

7-Monohydroxyethylrutoside was administered i.v. in 10 min at a dose of 1500 mg m^−2^ 60 min before every DOX administration. If cardiotoxicity would be observed in the first three evaluable patients, administration according to this dosing scheme would be changed and a following patient would receive DOX infusion either immediately after monoHER (because in plasma and heart, *C*_max_ of monoHER is obtained immediately after the end of infusion; Abou El Hassan *et al*, 2003; Willems *et al*, 2006) or with an interval of 2 h (to give monoHER the opportunity to convert into an active metabolite, if any). If cardiotoxicity was maintained in both patients, then the study would be finished. If one of the two patients shows cardioprotection, then 10 additional patients will be treated with the protecting scheme.

### Patient evaluation

Before starting, patients were evaluated by a full blood count, serum biochemistry including liver function tests, lactate dehydrogenase, and cholesterol. Risk factors for cardiovascular disease were also evaluated. An LVEF and an ECG were performed before entry into the study.

Every subsequent administration of monoHER and DOX was preceded by a full blood count, liver enzymes, serum creatinine and a routine 12-lead ECG. A complete blood count was also done 10 days after chemotherapy.

After a cumulative dose of 300 mg m^−2^ DOX, an endomyocardial biopsy was performed and the LVEF was measured. The latter was repeated at least 3 weeks after the last dose of DOX with a biopsy if possible. For logistic reasons, the biopsy of patient no. 8 was done after a cumulative dose of 375 mg m^−2^ DOX.

### Endomyocardial biopsy

During a left heart catheterisation, a 104 cm, seven french biopsy forceps was used to obtain tissue from the left ventricle. Three to four specimens 0.5–1 mm in diameter were obtained. The specimens were fixed in 4% buffered formaldehyde and prepared for electron microscopy.

### Histological analysis and biopsy scores

After fixation in 4% buffered formaldehyde, the heart tissue was post fixed in 1% osmium tetroxide. The tissue was then dehydrated through a graded series of ethanol solutions of 70–95% and embedded in JB-4 Plus resin. Thereafter, 0.5–3.0 *μ*m thick sections were cut with a glass knife. These semithin sections were processed for electron microscopy. Cardiomyocytes with >2 microvacuoles, macrovacuoles and/or loss of myofibrils were counted as deviant. The morphological grade determined from the specimens examined by electron microscopy was scored on a six-point scale previously described by Billingham *et al* (1978) (Bristow *et al*, 1982): in grade 0, cells are normal; in grade 1, 1.5, 2 and 2.5 deviant cells are <5, 5–15, 16–25 and 26–35%, respectively; in grade 3, cell damage is >35%.

### Off-study criteria

Patients went off study in case of progressive disease, a serious cardiac event or other events that precluded further treatment. Criteria described by Shapiro *et al* (1999) were used for diagnosis of cardiac events. Episodes of cardiac dysfunction were characterised according to the NYHA functional classification (Seidman *et al*, 2002).

### Assessment of tumour response

Assessment of tumour response was carried out every 2–3 cycles by CT scan, using standard ECOG criteria (Oken *et al*, 1982).

### Statistical analysis

This trial was an open-labelled, controlled study. We compared the data of our patients with those of 14 patients from the study of Torti *et al* (1986) treated with a cumulative dose of DOX between 200 and 300 mg m^−2^ alone using the *χ*^2^ test. Our hypothesis was that adding monoHER to DOX would eliminate its cardiotoxicity up to a cumulative dose of at least 300 mg m^−2^, which was the upper limit of the dose interval of DOX from Torti's patients. To achieve a power of 80%, 11 patients would be required. This sample size was obtained when applying the *χ*^2^ test with significance level 0.05 and assuming a response rate (i.e. no cardiac damage) in the experimental arm of 80%. If at least five patients show DOX-induced damage, statistical significance cannot be achieved anymore and the study should be stopped.

## RESULTS

Eight patients meeting the inclusion criteria were enrolled (Table 1).

Analysis of risk factors indicated that patient no. 6 was treated for hyperhomocysteinaemia. Two other patients had an elevated body mass index of 29.1 (no. 1) and 30.1 (no. 7), indicating overweight and obesity, respectively. Patient no. 2 had hyperlipidaemia (LDL of 6.3 mmol l^−1^; normal values ⩽5.0). None of the other patients had risk factors for cardiovascular disease. All patients were classified as NYHA class ^2^ and had a performance status (WHO) ⩽2.

Five patients received a cumulative dose of ⩾300 mg m^−2^ DOX and underwent a biopsy. In the other three patients, DOX was discontinued before this dose, due to progressive disease.

Patient no. 3 received irradiation after he was operated for a parapharyngeal tumour with a modified radical neck dissection at his right site because of a malignant peripheral nerve sheet tumour. Both patient nos. 7 and 8 received postoperative irradiation after resection of the malignant fibrous histiocytoma in their right upper leg.

### Treatment with DOX preceded by monoHER

During monoHER infusion, two patients reported adverse events. One patient described a sensation of fullness in his stomach, which developed during infusion of monoHER and disappeared soon after the end of the infusion. The other patient experienced itching in the skin of the neck during monoHER infusion and disappearing rapidly after the infusion. Both patients experienced the events during each cycle. A causal relationship cannot be excluded. The other six patients did not experience adverse events.

None of the patients had a delay in receiving subsequent cycles of chemotherapy. As expected, all patients developed chemotherapy-related leukopaenia, which recovered before the start of the next cycle. No disturbances of liver enzymes and serum creatinine were noted.

### Biopsy scores

**Figure 1:**
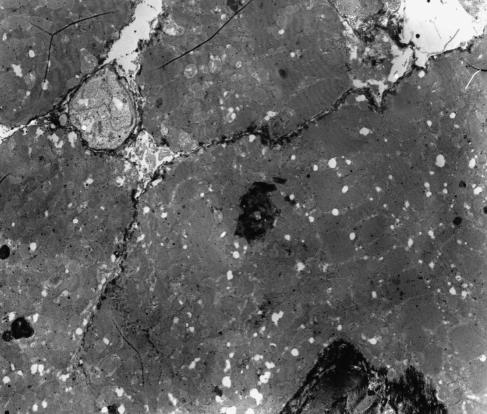
Heart tissue from patient no. 3 after 300 mg m^−2^ DOX. Evaluation by electron microscopy demonstrated an abundant presence of microvacuoles in the cardiomyocytes.

The endomyocardial biopsy scores are shown in Table 2. After 300 mg m^−2^, the first three evaluable patients showed abnormalities consistent with DOX-induced cardiotoxicity, that is the presence of microvacuoles dominated (Figure 1). In patient nos. 3 and 4, microvacuoles were present in 52 and 20% of the cardiomyocytes, respectively. In patient no. 6, 33% of the cardiomyocytes had >2 small vacuoles per cardiomyocyte. Because each of the first three evaluable patients showed cardiotoxicity, it was decided to stop this dosing scheme. The time interval between monoHER and DOX administration was changed and patient no. 7 received DOX immediately after monoHER. In the biopsy of this patient, microvacuolisation was observed in 60% of the cardiomyocytes. After this result, the interval was changed again and patient no. 8 received DOX 2 h after monoHER administration. In this patient, microvacuolisation was detected in 49% of the cardiomyocytes. After these results, the study had to be considered negative, because no cardioprotective effect of monoHER was observed in the five patients.

**Figure 2:**
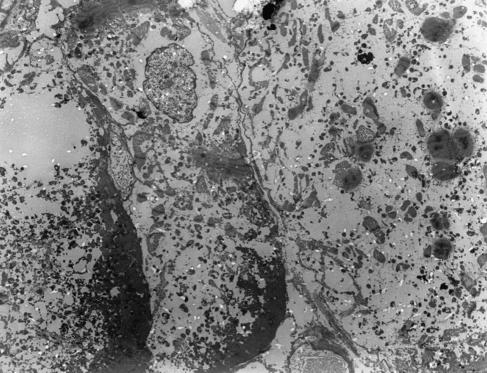
Heart tissue from patient no. 3 after 480 mg m^−2^ DOX. In addition to the vacuolisation, a loss of myofibrils is observed with electron microscopy.

Second biopsies were also taken after 450–480 mg m^−2^ of DOX in two patients (Table 3). The score of patient no. 4 had increased from 2.0 to 2.5, whereas the score of patient no. 3 remained 3. Striking was the increase in loss of myofibrils in addition to the microvacuoles observed in the cardiomyocytes after 300 mg m^−2^ (Figure 2).

Ten months after his last cycle of DOX, patient no. 4 underwent a third biopsy. This time, the cardiomyocytes showed recuperation of myofibrils, whereas the number of abnormal cardiac cells (microvacuolisation) was less than that in the first two biopsies (score 2).

No complications occurred during the seven biopsy procedures in the first four patients. However, the last patient (no. 8) developed a small pericardial effusion after the biopsy. This was preceded by chest pain and transient supraventricular rhythm disturbances. These sequelae disappeared within a few days and after a week she recovered and received the sixth cycle of DOX.

### Monitoring and evaluation of cardiac function

In all patients, the ECG remained unchanged during therapy and no cardiac dysfunction occurred.

The five patients who received at least 300 mg m^−2^ DOX started with an LVEF >50% (Table 2). After 300–375 mg m^−2^ DOX, the LVEF decreased in 4 out of 5 patients. This decrease was not related with the biopsy score or the time interval between monoHER and DOX infusions. Unfortunately, no information on the LVEF was available for patient no. 6.

After 480 mg m^−2^ DOX, the LVEF in patient no. 3 decreased to 53%, which is a decline of 25% compared to the LVEF before starting the study (71%). The patient had no symptoms of cardiac failure. Ten months after his last cycle of chemotherapy, the LVEF remained stable and he remained without cardiac symptoms.

The LVEF of patient no. 4 showed an initial drop from 72% before starting chemotherapy to 60% after 300 mg m^−2^ DOX (decline of >15%). This value remained stable up to 450 mg m^−2^ of DOX, and at 10-month follow-up the patient still had an excellent physical condition.

### Evaluation of tumour response

Response to the chemotherapy was remarkable in the four patients with metastatic soft-tissue sarcoma (STS). Three of them developed a partial remission (PR), as observed on the CT scan (Table 1). In two of these patients (nos. 4 and 7), PR was maintained up to the present, that is, 30 and 16 months after the start of chemotherapy, respectively. The other patient (no. 3) had progressive disease after a PR of 9 months duration. The fourth patient (no. 8) achieved stable disease for at least 7 months, while continuing therapy with DOX up to a cumulative dose of 495 mg m^−2^.

## DISCUSSION

On the basis of the promising results with monoHER observed in preclinical experiments, we performed the present phase II study in patients with metastatic cancer. The cardioprotective effect of monoHER on DOX-induced cardiotoxicity was evaluated by endomyocardial biopsy. However, the results indicated that the preclinical observations were not translated into protection against DOX-induced heart damage in humans.

The golden standard for early detection of DOX-induced cardiotoxicity is the endomyocardial biopsy, because of its high sensitivity and high specificity (Torti *et al*, 1986). Currently, the most common method used to detect DOX-induced cardiac damage is the evaluation of the LVEF, but usually at later stages (Kilickap *et al*, 2005; Villani *et al*, 2006). For detection of cardiotoxicity at an earlier stage, the use of biochemical markers such as atrial and brain natriuretic peptides, endothelin-1 and also cardiac troponin-T and -I have been investigated (Yamashita *et al*, 1995; Suzuki *et al*, 1998; Kilickap *et al*, 2005). However, large-scale studies addressing whether these biomarkers following DOX treatment will be predictive for the development of late-onset heart failure are lacking. Therefore, despite its invasiveness, we chose the endomyocardial biopsy for this evaluation.

Data of Billingham *et al* (1978) showed that anthracycline-induced myocardial damage occurred in nearly all patients treated with cumulative DOX doses of 240 mg m^−2^. Therefore, we could expect detectable heart damage after a cumulative dose of 300 mg m^−2^, which would allow the registration of protection by monoHER. However, all five patients undergoing the biopsy procedure after a median cumulative dose of 300 mg m^−2^ showed DOX-induced cardiotoxicity with a mean biopsy score of 2.7 according to Billingham *et al* (1978). This score was independent of the time interval between monoHER and DOX infusion and much higher than the biopsy score of 1.4, which is expected after a cumulative dose of 300 mg m^−2^ DOX according to the linear regression analysis of Torti *et al* (1986). In our patients, mainly one of the three morphological changes, that is, small vacuoles of varying size, was observed after 300 mg m^−2^ DOX, whereas in Torti *et al*'s study also partial or total myofibrillar loss was observed. However, in Torti's study, it is not clear after which dose this occurred. In addition to microvacuolisation, loss of myofibrils was demonstrated in two patients after higher cumulative doses of DOX. This suggests that the occurrence of microvacuoles as such is a marker of cytotoxicity of DOX treatment. Billingham *et al* (1977) described that these microvacuoles appear early as a swelling of the sarcoplasmic reticulum, which eventually coalesce to form large spaces in the cytoplasm (macrovacuoles). The possible change from microvacuoles into macrovacuoles in time is not clear from literature.

Thus, instead of protection, our patients had higher biopsy scores than the historical controls. However, it should be noted that the data of Torti *et al* (1986) were obtained after a cumulative dose of 200–300 mg m^−2^ with biopsies from the right ventricle, whereas biopsies from our patients were obtained after a cumulative dose of 300 mg m^−2^ from the left ventricle. Previously, it was shown that ultrastructural abnormalities of cardiomyocytes were more pronounced in biopsy specimens from the left ventricle than those from the right ventricle (Mortensen *et al*, 1986). In addition to this, there is a considerable variability in patient sensitivity to the cardiotoxic effects of DOX (Ferrans 1978; Mason *et al*, 1978). These data may explain a possible overestimation of the toxic effect of DOX on the heart tissue in our patients. Although heart failure is directly related to the degree of myocyte damage (Bristow *et al*, 1978), none of our patients developed heart failure, although the LVEF dropped by 16% in two patients, whereas it decreased in two other patients by only 4–6% (in comparison to the initial LVEF).

In the third biopsy procedure of patient no. 4, the score of the cardiac tissue had improved. A recuperation of myofibrils in the cardiomyocytes was observed and the microvacuolisation was less. These findings are in agreement with a previous study reporting that some improvement of the histological damage may occur (Mackay *et al*, 1994). This is in contrast with other results, which indicated that DOX-induced cardiotoxicity is an ongoing progressive process (Billingham *et al*, 1978).

The contrasting effects of monoHER found in animal and human studies may be attributed to differences in metabolism between the species. Therefore, patient no. 8 was treated with a 2 h interval. However, this interval change did not reduce DOX-induced cardiotoxicity. In addition, no metabolites of monoHER have been detected until now. On the other hand, it cannot be excluded that during scavenging of the reactive oxygen species, the antioxidant monoHER is converted into a reactive oxidation product, which, like the oxidation product of quercetin, may be prone to form adducts with thiol groups from glutathione and proteins (Boots *et al*, 2005a). Depletion of glutathione may in addition to the low antioxidant status of the cardiomyocyte (Nowak and Drzwoski, 1996) reduce cardioprotection, while monoHER-protein adducts may cause additional toxicity (Boots *et al*, 2005b).

Another unexpected observation in our study was that three of the four patients with STS had objective remissions, while the fourth patient had stable disease. Normally, objective responses on DOX in STS patients without prior chemotherapy are approximately 25% (Santoro *et al*, 1995). Although our observation was done in a very limited number of patients, our result is much better than expected, because the chance of observing an objective response in four consecutive STS patients treated with DOX is 0.4%. Thus, it is suggestive that monoHER enhances the antitumour activity of DOX in STS.

This observation is in agreement with potentiating antitumour effects of a few flavonoids observed *in vitro* (Elangovan *et al*, 1994; Sliutz *et al*, 1996; Debes *et al*, 2003). The background for this effect may be that the concentration of GSH may play a role in STS chemoresistance (Hochwald *et al*, 1997) and that GSH depletion may increase the antitumour efficacy (Siemann and Beyers, 1997). Thus, the same mechanism may play a role as hypothesised for the cardiomyocytes.

As a consequence of the above-mentioned aspects, there may be a dose-depending transition in the effect of monoHER, that is a high dose (⩾1500 mg m^−2^) for obtaining a potentiating effect of the antitumour effect for at least STS and a low dose (somewhere below 1500 mg m^−2^) for obtaining cardioprotection. These aspects have to be elucidated further in the near future. It may be concluded that monoHER at a dose of 1500 mg m^−2^ did not protect against DOX-induced cardiotoxicity in patients with metastatic disease, but may have an enhancing effect on the antitumour activity of DOX in patients with metastatic STS. Further preclinical and clinical investigations seem to be warranted to investigate the postulated dose-depending transitional effect of monoHER.

## Figures and Tables

**Table 1:** Patient characteristics

**Patient no.**	**Age/sex**	**Diagnosis**	**Total dose of DOX (mg m^−2^)**	**Response on DOX**	**Biopsy**
01	62/F	Breast cancer	100	PD	N
02	54/F	Adrenal cortical cancer	150	PD	N
03	25/M	Malignant peripheral nerve sheet tumour	480	PR	Y
04	64/M	Malignant fibrous histiocytoma	450	PR	Y
05	55/F	Breast cancer	100	PD	N
06	48/F	Breast cancer	300	SD	Y
07	45/F	Malignant fibrous histiocytoma	300	PR	Y
08	56/F	Malignant fibrous histiocytoma	375	SD	Y

Age in years; sex F female, M male; assessment of tumour response was done by using standard ECOG criteria (PD=progressive disease; PR=partial remission; SD=stable disease); Y=yes; N=no.

**Table 2:** Patients who received at least a cumulative dose (cum. dose) of 300 mg m^2^ DOX and underwent an endomyocardial biopsy

**Patient no.**	**Biopsy score cum. dose (no. of cycles × dose per cycle, time of bolus infusion/Δ*t*/grade**	**LVEF before dox**	**LVEF after ⩾300 mg m^−2^**
03	300 (4 × 75, 15′)/60/grade 3	71%	67%
04	300 (4 × 75, 15′)/60/grade 2	72%	60%
06	300 (6 × 50, 15′)/60/grade 2.5	52%	ND
07	300 (4 × 75, 15′)/10/grade 3	75%	63%
08	375 (5 × 75, 15′)/120/grade 3	65%	63%

Δ*t*=time between end of monoHER infusion and start of DOX infusion in minutes.

The morphological grade was scored on a six-point scale previously described by Billingham and Bristow; LVEF=left ventricular ejection fraction.

**Table 3:** Two patients who underwent an extra endomyocardial biopsy at >300 mg m^−2^ of DOX

**Patient no.**	**Cum.dose/biopsy score/LVEF**	**Biopsy score/LVEF 10 months after the last cycle**
03	480/grade 3/53%	ND/51%
04	450/grade 2.5/66%	Grade 2/62%

The morphological grade was scored on a six-point scale previously described by Billingham and Bristow; LVEF=left ventricular ejection fraction; ND=not done.
